# Minimally invasive left colectomy with total intracorporeal anastomosis versus extracorporeal anastomosis. A single center cohort study. Stage 2b IDEAL framework for evaluating surgical innovation

**DOI:** 10.1007/s00423-024-03387-9

**Published:** 2024-07-19

**Authors:** Xavier Serra-Aracil, Irene Gómez-Torres, Andrea Torrecilla-Portoles, Anna Serracant-Barrera, Albert García-Nalda, Anna Pallisera-Lloveras

**Affiliations:** 1 Department of Surgery, Autonomous University of Barcelona, Parc Tauli s/n, Sabadell, Barcelona, 08208 Spain; 2 Coloproctology Unit, General and Digestive Surgery Service, Parc Tauli Institute for Research and Innovation I3PT, Parc Tauli University Hospital, Sabadell, Spain

**Keywords:** Intracorporeal anastomosis, Left colectomy, Robotic colectomy, Robotic left colectomy, Minimally invasive left colectomy

## Abstract

**Purpose:**

Performing intracorporeal anastomoses in minimally invasive colon surgery appears to provide better short-term outcomes for patients with colon cancer. The aim of the study is to compare surgical aspects and short-term outcomes between intracorporeal and extracorporeal techniques in left colectomies with both laparoscopic and robotic approaches and evaluate advantages and disadvantages of intracorporeal anastomosis according to IDEAL framework (Exploration, stage 2b).

**Methods:**

This is a single center, ambispective cohort study comparing total intracorporeal anastomosis (TIA) and standard surgery with extracorporeal anastomosis (EA). Patients with colon cancer treated by left colectomy, sigmoidectomy and high anterior resection by total intracorporeal anastomosis between May 2020 and January 2023 without exclusion criteria were prospectively included in a standardized database. Short-term outcomes in the group undergoing TIA were compared with a historical EA cohort. The main assessment outcomes were intraoperative complications, postoperative morbidity according to the Clavien-Dindo scale and the comparison of pathological. We conducted a preliminary comparative study within the TIA group between approaches, a primary analysis between the two anastomotic techniques, and a propensity score matched analysis including only the laparoscopic approach, between both anastomotic techniques.

**Results:**

Two hundred and forty-six patients were included: 103 who underwent TIA, 35 of them with laparoscopic approach and 68 with robotic approach, and a comparison group comprising another 103 eligible consecutive patients who underwent laparoscopic EA. There were no statistically significant differences between the two groups in terms of demographic variables. No statistically significant differences were observed in anastomotic dehiscence. Intraoperative complications are fewer in the TIA group, with a higher C-Reactive Protein levels. Relevant anastomotic bleeding and the number of retrieved lymph nodes were higher in EA group. Nevertheless, no differences were observed in terms of overall morbidity.

**Conclusion:**

Minimally invasive left colectomy with intracorporeal resection and anastomosis is technically feasible and safe suing either a laparoscopic or a robotic approach. Clinical data from this cohort demonstrate outcomes comparable to those achieved through the conventional EA procedure in relation to postoperative morbidity and oncological efficacy, with indications suggesting that the utilization of robotic-assisted techniques may play a contributing role in enhancing overall treatment outcomes.

## Introduction

Minimally invasive surgery has represented a major step forward in the treatment of colorectal cancer. Compared to open surgery, laparoscopic approach has provided better patient recovery and better short-term outcomes, such as lower rates of postoperative complications, less postoperative pain with smaller incisions and less surgical aggression, lower mortality at 30 days after surgery, lower blood loss, and shorter hospital stay [1–4]. 

Today, laparoscopy is the preferred approach in colorectal surgery, firmly established in Western countries. For transit reconstruction anastomosis, extracorporeal anastomosis (EA) is commonly employed, involving tissue extraction through an accessory incision. Mobilizing the colon is essential to prevent unwanted traction on the mesocolon and mitigate risks of ischemia or bleeding. Recently, intracorporeal anastomosis (IA) techniques have emerged to enhance results and minimize tissue manipulation, avoiding excessive traction [5, 6]. 

Most comparisons of IA and EA techniques have focused mainly on surgery of the right colon and the mid or low rectum. Multiple studies have shown the benefits of IA, such as shorter hospital stay, lower postoperative complications, faster gastrointestinal recovery, and more accurate lymphatic resection for oncological assessments [7, 8]. 

The literature on intracorporeal resection and anastomosis in left colon and sigmoid surgery is limited. In recent years, various techniques have been described in both laparoscopic and robotic surgery, promising good short- and long-term outcomes [9–16]. 

However, comparing these IA techniques directly to the standard extracorporeal method is complex. The literature’s findings on functional disparities between various anastomosis types are inconsistent. While side-to-end and side-to-side anastomoses are considered safe alternatives to end-to-end anastomosis, mechanical end-to-end anastomosis yields superior functionality with the lowest complication rates [17, 18].

Currently, the clinical use of IA in left colectomy is limited due to insufficient evidence supporting its superiority over extracorporeal anastomosis. The lack of randomized trials and the considerable data heterogeneity contribute to this limitation.

Robotic surgery has introduced new IA techniques, leveraging its benefits such as enhanced visualization, precise dissection, reduced tremor, and increased wrist flexibility for intracorporeal sutures.

In left colectomy, some studies suggest the robotic approach can reduce morbidity, conversion rates, and hospital stays. However, not all studies corroborate these benefits [19–22].

Regarding robotic IA, literature primarily concentrates on right-sided techniques [23, 24], while left-sided anastomoses are often extracorporeal. Limited studies exist on robotically performed IA. Recent research indicates that robotic left colectomy with entirely intracorporeal anastomosis (R-TIA) is safe and feasible, yielding positive short-term outcomes. In terms of comparisons with the laparoscopic approach, there are few but promising findings for R-TIA [25–34].

Our hypothesis is that IA in minimally invasive left colon, sigmoid, and upper rectum surgery is non inferior to the standard extracorporeal techniques. The main objective is to determine the non-inferiority of the intracorporeal technique regarding the primary variable, anastomotic dehiscence. The secondary objectives include the comparison of both techniques with respect to short-term outcomes, considering intraoperative results, postoperative complications, hospital stay, and pathology. Additionally, it includes assessing differences between laparoscopic and robotic IA and their impact on outcomes compared to EA. This study corresponds to IDEAL framework Stage 2B (Exploration).

The ultimate goal of our research process on intracorporeal anastomosis of the left colon, sigmoid colon, and upper rectum is to define the intervention, its indications, and the quality standards by means of a prospective cohort study in order to address and overcome the obstacles to a definitive comparative trial [35–37]. 

## Materials and methods

### Study design

This is a single center, ambispective cohort study of consecutive patients undergoing laparoscopic or robotic left colectomy, sigmoidectomy, and high anterior resection for colorectal adenocarcinoma. A retrospective cohort of patients who had undergone left colectomy with conventional EA with laparoscopic approach was used as a control group for comparison with the prospective cohort undergoing TIA, either laparoscopic or robotic. Both groups comprised 103 patients.

### Study population. Selection criteria

All patients were evaluated by the multidisciplinary colorectal tumor committee. The therapeutic strategy was determined in accordance with the Colon Cancer NCCN Clinical Practice Guidelines in Oncology. Version 2. 2021 [38].

#### Inclusion criteria

Patients over 18 years of age diagnosed with colon adenocarcinoma confirmed by biopsy, located in left colon, sigmoid colon or upper third of the rectum above the peritoneal reflection, who underwent a left colectomy, sigmoidectomy or upper anterior resection with inferior mesenteric vessels resection and middle colic artery preservation, minimally invasive laparoscopic or robotic approach; consent to undergo the procedure; aim of R0 resection and surgery with curative intent.

#### Exclusion criteria

Refusal to participate in the study; non-curative intent surgery (palliative surgery); emergency surgery; synchronous tumors, multivisceral resections, malnutrition (preoperative albumin ≤ 3.4 g/dl); pregnancy or pulmonary disease that precluded the creation of the pneumoperitoneum.

Patients were included in the study consecutively from the time of inclusion of the first eligible patient.

### Perioperative management

The study followed the Ethical Principles for Medical Research Involving Human Subjects as outlined in the Declaration of Helsinki. The local Institutional Ethics Committee approved the use of the intracorporeal technique for the treatment of tumors and diverticular disease of the left colon, sigmoid, and upper third of the rectum (CEIC 2020/679). The study protocol was registered at www.ClinicalTrials.gov (NCT0445693). The STROBE guidelines for observational studies were followed [39]. Informed consent was obtained from the patients after an explanation of the risks and benefits of the procedure.

The baseline characteristics of the patients were collected, including age, sex, ASA score (American Society of Anesthesiologists), body mass index (BMI), and main diagnosis.

All patients who met the selection criteria were included in the study. Within the scheduled surgery, all patients followed the prehabilitation program, where they try to optimize their preoperative condition. Abdominal CT and a routine blood test were performed. All patients underwent antegrade mechanical preparation. Oral antibiotics were administered: erythromycin 1 g and neomycin 1 g (3 doses the day before surgery), antibiotic prophylaxis (amoxicillin-clavulanate Ac 2 g / ev or, in the case of allergy, metronidazole 1 g/ev and gentamicin 3–5 mg/kg/ev) during anesthetic induction and thromboembolic prophylaxis (enoxaparin 40 mg administered subcutaneously) according to institutional protocol. Since April 2020, all prospective surgical patients underwent a COVID19 PCR test and a chest CT in the 48 h prior to surgery. A positive result contraindicates surgery.

All surgical procedures were performed by the colorectal surgery team at Parc Taulí University Hospital, who have extensive experience in minimally invasive colorectal surgery. The team comprises five surgeons.

The surgical procedure was conducted under general anesthesia with endotracheal intubation and catheterization of the bladder. A standardized enhanced recovery after surgery (ERAS) protocol [40] was applied to manage all patients. Early mobilization and a liquid trial commenced 6 h post-surgery. Uniform analgesic measures were administered to all patients. Oncological surgeries adhered to prevailing guidelines and established oncological criteria.

### Surgical technique (Fig. 1) [11]

In the historical cohort of patients operated upon using the conventional technique, the approach was laparoscopic. In the prospective cohort of patients who underwent TIA, the approach used was determined by the availability of robotic equipment, as this resource is also used by other surgery teams at our institution. Consequently, both laparoscopic and robotic approaches were applied in this cohort. IA techniques with both laparoscopic and robotic approaches have been described in previous work [10–12]. 

**Fig. 1:**
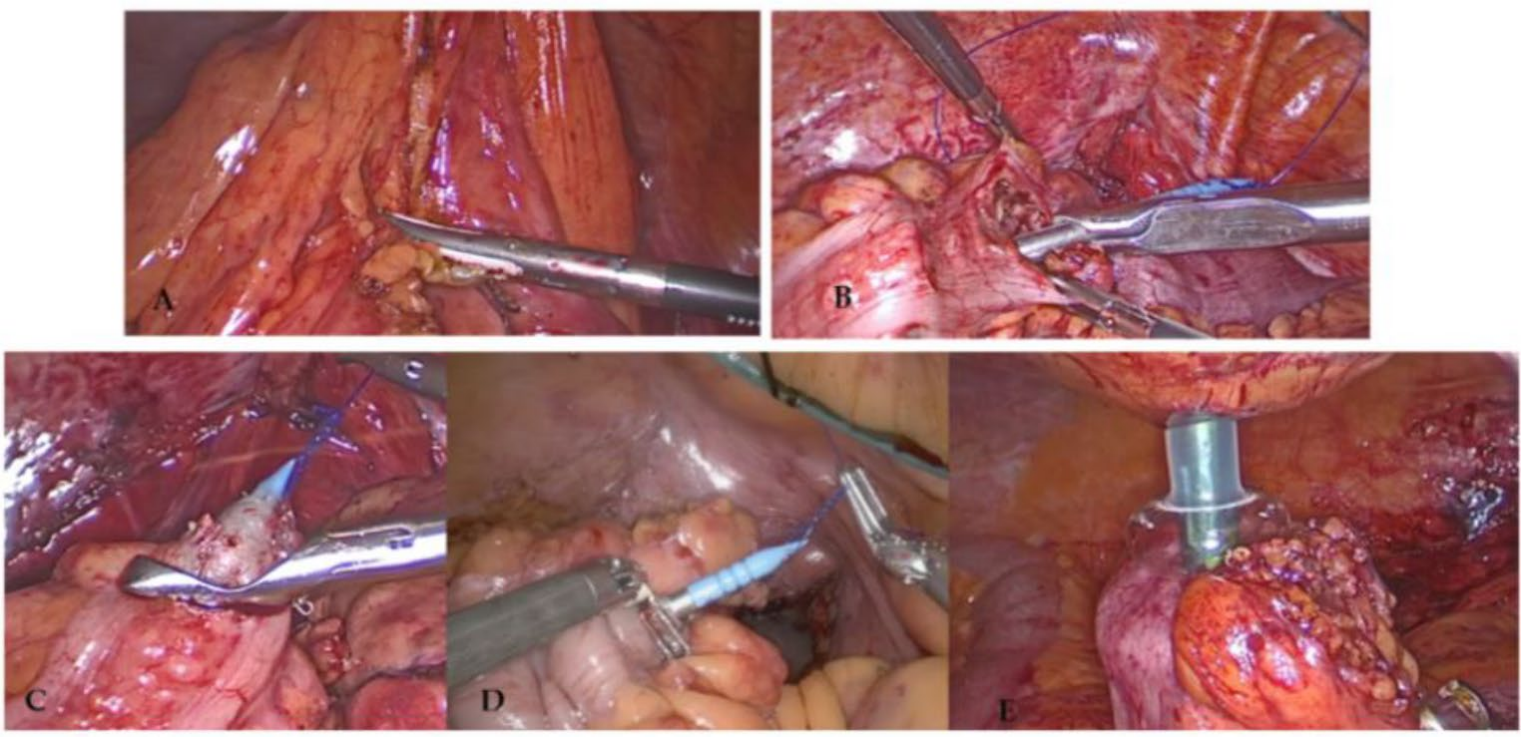
Surgical technique. **A** Mesocolon section using the IMA as a reference. **B** Colotomy and stapler anvil head placement. **C** Exteriorization of the stapler head pulling from prolene suture. **D, E** Removal of the anvil tip [11]

Patients underwent a left colectomy, sigmoidectomy, or high anterior resection with laparoscopic approach in accordance with oncological criteria for lymphadenectomy and vascular sectioning and a 29-mm curved circular stapler (B. Braun, Melsungen, Germany) was used in both cohorts. ICG was used but not systematically.

#### a. Access and Placement (Fig. 2)

Laparoscopic Approach: Umbilical Hasson port for the camera, a 12 mm port in the right iliac fossa, and 5 mm ports in the right upper quadrant and left iliac fossa.

**Fig. 2:**
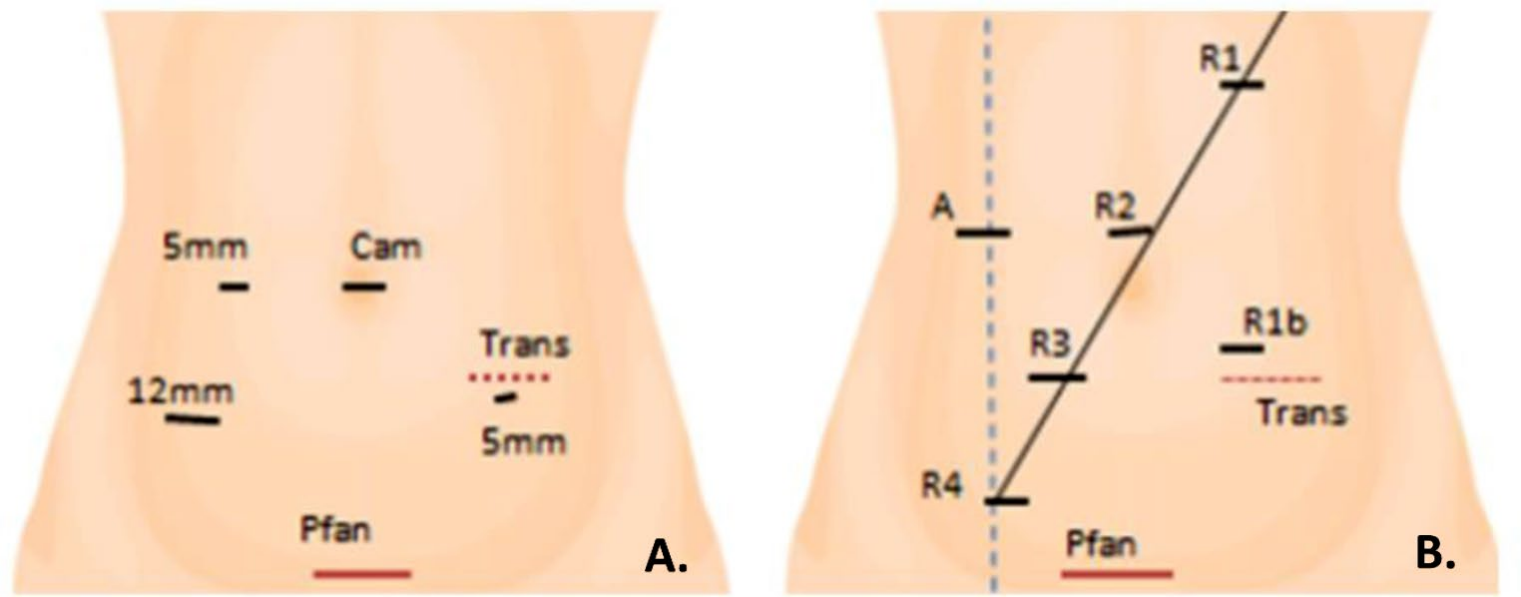
Diagram of trocar placement and possible accessory incisions for specimen extraction (Pfannenstiel incision or transverse incision depending on the patient’s characteristics). **A** Laparoscopic approach. **B** Robotic approach

Robotic Approach: Use 12 mm (R3) and 8 mm (R1, R2, R4) robotic ports following the left colon technique. An extra 12 mm port (R1b) if needed. Da Vinci Xi Robot was docked on the left side.

#### b. Patient positioning

The patient is placed in a supine with arms tucked and legs in modified lithotomy position. The patient is tilted to the right and placed in a Trendelenburg position for both approaches.

#### c. Dissection

The inferior mesenteric pedicle is divided while preserving the ureter and autonomic nerves. High ligation of the inferior mesenteric artery (IMA) is performed, along with standardized mobilization of the splenic flexure if necessary. Subsequently, the colon is mobilized, and the upper rectum and mesorectum are transected using a linear stapler.

#### d. Proximal Colon transection and Anastomosis

All patients undergo end-to-end anastomosis.

1. Extracorporeal Anastomosis Technique (EA - Historical Cohort): The sigmoid adhesions are separated, and the rectosigmoid junction is mobilized. The distal colon is sectioned using an Echelon stapler. A Pfannenstiel incision is made to exteriorize the tumor. Subsequently, an extracorporeal resection of the left mesocolon/mesosigma is performed, and the anvil of a 29-mm curved circular stapler (B. Braun, Melsungen, Germany) is placed. Finally, pneumoperitoneum is reestablished for colorectal anastomosis.
2. Totally Intracorporeal Anastomosis Technique (TIA - Prospective Cohort)
  2.1 Laparoscopic Left Colectomy with TIA:
    An additional incision is made for the insertion of the head of a 29-mm curved circular stapler (B. Braun, Melsungen, Germany), secured with a 0 Prolene® suture and protected with an endoscopic bag. Intracorporeal resection of the left mesocolon is performed up to the proximal end, using the vascular pedicle as a reference. The intracorporeal insertion of the stapler head and proximal transection is carried out by introducing the stapler head through the colotomy, guiding the removal of the stapler head through the colon wall using a prolene suture, and performing the distal section with an Echelon stapler (60 mm blue load). The stapler head is exteriorized at the proximal end of the colon, and a purse-string suture is performed with 2.0 prolene.
  2.2 Robotic Left Colectomy with TIA:
    An accessory incision is made to introduce the head of the anvil with the tip into the abdominal cavity. Intracorporeal resection of the left mesocolon is carried out similarly to the laparoscopic approach after robot docking. Next, an end-to-end mechanical anastomosis is fashioned using a 29 mm Braun circular stapler, verifying the anastomotic rings and conducting an air test.
  Four to six reinforcing stitches with 2 − 0 silk are placed in all patients, regardless of the type of anastomosis or the type of approach (laparoscopic and robotic).

#### e. Piece extraction

An endoscopic bag is used to extract the specimen. In the EA group, extraction is done through the Pfannenstiel incision. In the TIA group, extraction is performed through the same point of stapler head introduction, this technique allows you to choose the site of the minilaparotomy (a 3–5 cm Pfannenstiel incision or a transverse mini-laparotomy, depending on the patient’s anatomical characteristics), with protection using the dual-ring retractor Alexis O Wound Protector C8401.

### Outcome measures

Primary variable Anastomotic leakage (AL), defined by Peel et al.

Secondary outcome variables: Surgical aspects included surgery type, operative time, intraoperative complications (unexpected adverse events during surgery), and conversion to open surgery. Postoperative factors within 30 days encompassed overall morbidity, Clavien-Dindo morbidity categorization, low morbidity (Clavien-Dindo ≤ II), high morbidity (Clavien-Dindo > II), Comprehensive Complication Index (CCI), surgical site infection (SSI), according to the Center for Disease Control (CDC) National Surveillance System for Nosocomial Infections, post-operative ileus, ICU admission, relevant anastomotic bleeding, time to first meal, CRP levels, surgical and non-surgical complications, length of stay, return to operating room (OR), return to emergency department (ED), and postoperative mortality. Pathological variables involved pT, pN, tumor size, nodes identified, and the proportion of specimens with more than twelve nodes [41, 42]. 

Conversion to open surgery was considered if a midline laparotomy was performed or a Pfannenstiel incision greater than 10 cm was necessary. Tumors were staged in accordance with the UICC and TNM of the AJCC, 8th edition [43]. 

### Statistical analysis

The sample size of the comparative cohort study was calculated according to the primary outcome (AL) with non-inferiority criteria. Assuming a rate of AL of 7%, as expected in the standard extracorporeal treatment (historical cohort) vs. 5% with the intracorporeal technique, with a delta margin of no more than 8%, and a probability of erroneously rejecting the null hypothesis (one-sided alpha risk) of 0.05 and a probability of erroneously rejecting the alternative hypothesis (beta risk) of 0.2, we obtain, with an estimated 10% loss per group, a minimum figure of 74 patients per group.

Statistical analysis was performed with the SPSS version 29 (IBM Corporation, Armonk, NY). The prospective data collection permitted an analysis without missing values. Quantitative variables were depicted using mean values and standard deviation when normally distributed; otherwise, median, interquartile range, and range values were utilized. Categorical variables were presented as absolute numbers and percentages. Univariate statistical analysis of quantitative variables involving independent groups employed the Student t-test for parametric assessment or the Mann-Whitney non-parametric test as required. For categorical variables, Pearson’s X^2^ test or Fisher’s exact test was used, depending on the conditions. A p value < 0.05 was considered statistically significant, with a confidence interval of 95%.

### Propensity-score-matched analysis (PSM)

We conducted Propensity Score Matching (PSM) exclusively with the laparoscopic approach patients, to eliminate confounding variables and address potential biases in patient selection, arising from the assignment of patients to study groups or robotic approach. Propensity scores for all patients were determined based on the following factors: age, gender, ASA Score, prior abdominal surgery, surgery type and tumor size. Then we conducted a matching with 2:1 ratio using a caliper width of 0.1. The result is the pairing of two patients from the control group (EA group) with one patient from the prospective group (TIA group), based on the propensity score. This comparison was subsequently used in our subsequent analyses.

## Results

From May 2020 to January 2023, 247 patients were assessed for eligibility. Prospectively, a left colectomy, sigmoidectomy, or high anterior resection with TIA was performed in 129 patients. Twenty-six patients who met exclusion criteria were not included. Finally, the group comprised 103 patients (35 laparoscopic, L-TIA, and 68 robotic, R-TIA).

**Fig. 3:**
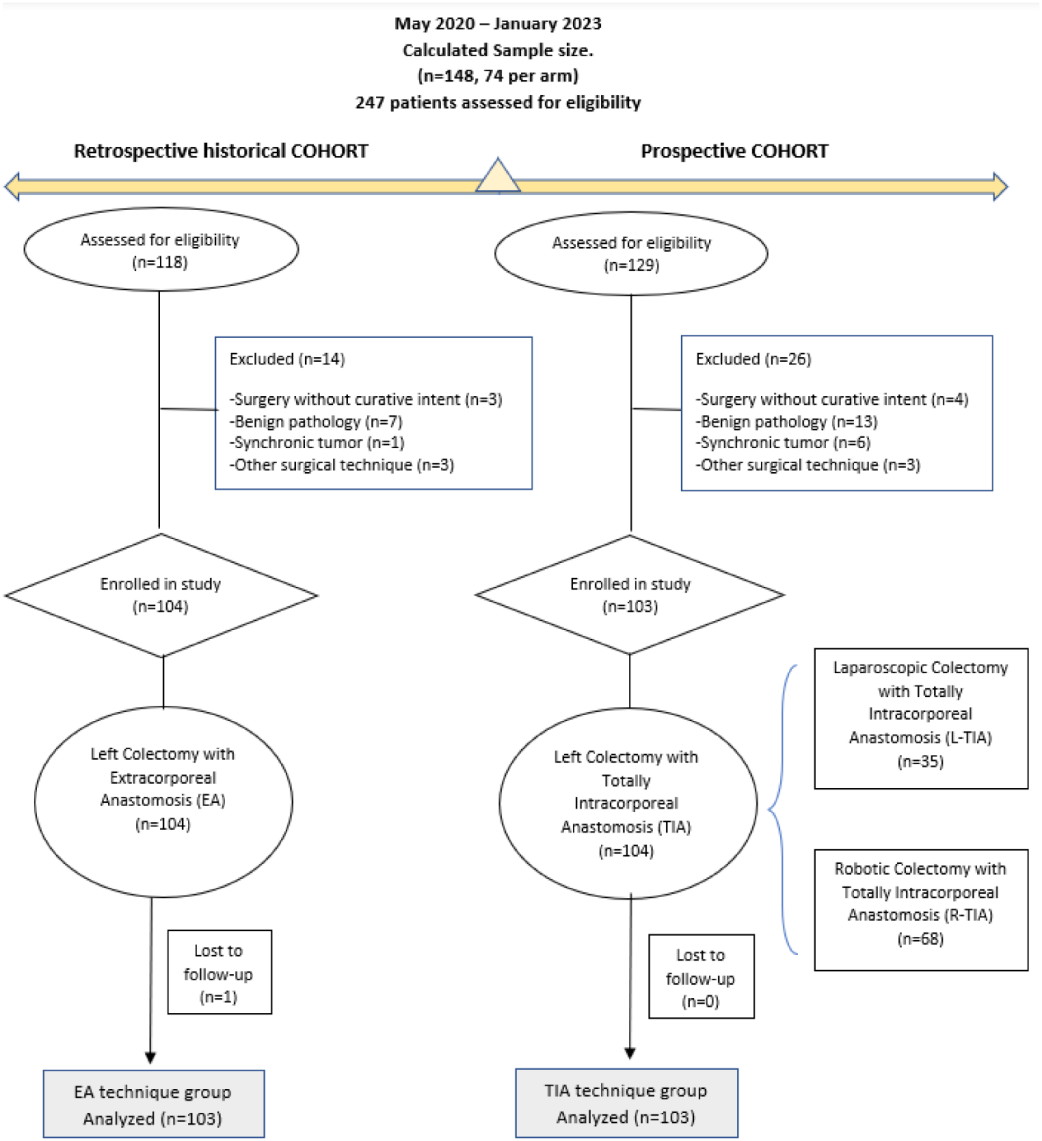
Cohort study flow diagram

In the historical cohort with the standard technique (EA), 118 patients who had undergone surgery immediately before the start of the prospective cohort were retrospectively collected. Finally, 103 patients were included without exclusion criteria. Figure 3 shows the cohort study flow diagram.

### Comparative study inside the TIA group: laparoscopic versus Robotic Approach

No significant differences were found in the primary outcome anastomotic leakage (2 cases vs. 2 cases, *p* = 0.60 (2.77, -5.90 to 11.45) or anastomotic leakage with surgical treatment (1 case vs. 0 cases, *p* = 0.34 (2.86, -2.66 to 8.38).

Operating time was significantly longer in the robotic approach group (L-TIA group 190 min, R-TIA group 217.5 min, *p* = 0.004). No significant differences were found in terms of morbidity or mortality, or in other postoperative features such as time to first meal, CRP levels on postoperative days 1 and 3, length of stay, or oncologic outcomes (Tables 1, 2 and 3).

**Table 1:** Patients’ characteristics and perioperative data in TIA group

Characteristic		Laparoscopic-TIA group (*n* = 35)	Robotic-TIA group (*n* = 68)	*p*- value (95% CI)
Sex, male/female, n (%)		19 (54,29%)/16 (45,61%)	36 (52,9%)/ 32 (47,1%)	0.89 (1,34, -18.98 to 20.33)
Age, median (IQR), years		69 (16)	71 (15)	0.99
BMI, median (IQR), kg/m2		26,22 (6,33)	27.57 (7,77)	0.53
ASA score (average), n (%)	I	2 (5,7%)	9 (13,2%)	0.56
	II	15 (42,9%)	32 (47,1%)	
	III	16 (45,7%)	24 (35,3%)	
	IV	2 (5,7%)	3 (4,4%)	
ASA score ≥ III, n (%)		18 (51,4%)	27 (39.7%)	0.25 (11,72, -8.51 to 31.96)
Surgical technique	Left colectomy	7 (20,0%)	9 (13,2%)	0.59
	Sigmoidectomy	16 (45,7%)	37 (54,4%)	
	High anterior resection	12 (34,3%)	22 (32,4%)	
Previous abdominal surgery, n (%)		18 (51,4%)	31 (45,6%)	0.57 (5.84, -14.51 to 26.19)
Operating time, median (IQR), min		190 (60)	217,5 (74)	0.004
Splenic flexure mobilization, n (%)		9 (25,7%)	21 (30,9%)	0.58 (-5,17, -23.34 to 18.17)
Intraoperative complications, n (%)		4 (11,4%)	8 (11,8%)	0.61 (-0,34, -13.36 to 12.69)
Conversion to open surgery, n (%)		0	0	1

Laparoscopic-TIA: Laparoscopic approach with totally intracorporeal anastomosis, Robotic-TIA: Robotic approach with totally intracorporeal anastomosis, BMI: Body Mass Index, ASA: American Society of Anesthesiologists, IQR: Interquartile range

**Table 2:** Thirty-day postoperative morbidity-mortality in intracorporeal technique patients in TIA group

Characteristic		Laparoscopic-TIA (*n* = 35)	Robotic-TIA (*n* = 68)	*p*-value (95% CI)
Overall morbidity, n (%)		13 (37,1%)	17 (25%)	0.14 (13.61, -5.30 to 32.53)
Clavien-Dindo classification (Cl-D)	0	22 (62,9%)	51 (75,0%)	0.31
	I	5 (14,3%)	9 (13,2%)	
	II	4 (11,4%)	6 (8,8%)	
	IIIa	0	0	
	IIIb	4 (11,4%)	2 (2,9%)	
	IVa	0	0	
	IVb	0	0	
	V	0	0	
Low-grade morbidity (Cl-D ≤ II), n (%)		9 (25,7%)	15 (22,1%)	0.67 (3,66, -13,86 to 21,17)
High-grade morbidity (Cl–D > II), n (%)		4 (11,4%)	2 (2,9%)	0.17 (8.99, -4.53 to 22.5)
Comprehensive Complication Index (CCI) score, median (IQR)		0 (8,7)	0 (6,5)	0.133
Surgical site infection (SSI), n (%)		4 (11,4%)	3 (4,4%)	0.22 (7.02, -4.6 to 18.63)
Incisional-SSI, n (%)		0	1 (1,5%)	0.66 (-1,47, -4,33 to 1,39)
Organ/space-SSI, n (%)		3 (8,6%)	2 (2,9%)	0.33 (5,63, -4,48 to 15,74)
Anastomotic leak, n (%)		2 (5,7%)	2 (2,9%)	0.60 (2.77, -5.90 to 11.45)
Surgical anastomotic leak, n (%)		1 (2,9%)	0 (0)	0.34 (2.86, -2.66 to 8.38)
Surgical complications, n (%)		11 (31,4%)	16 (23,5%)	0.47 (7.90, -10.49 to 26.29)
Non-surgical complications, n (%)		5 (14,3%)	3 (4,4%)	0.11 (9.87, -2.70 to 22.45)
Post-operative ileus, n (%)		3 (8.6%)	4 (5.9%)	0.68 (2.69, -8.14 to 13.52)
ICU admission, n (%)		1 (2.9%)	0 (0)	0.34 (2.86, -2.66 to 5.52)
Relevant anastomotic bleeding, n (%)		2 (5.7%)	5 (7.4%)	0.55 (-1.64, -11.52 to 8.24)
Time to 1st meal, median (IQR), h		6 (0)	6 (0)	0.84
1st day CRP, median (IQR)		3.35 (4.86)	3.56 (3,51)	0.50
3rd day CRP, median (IQR)		4,11 (7,44)	4.09 (6,71)	0.69
Returns to OR, n (%)		4 (11.4%)	2 (2.9%)	0.17 (8.49, -2.79 to 19.77)
Return to ED 30 days, n (%)		2 (5,7%)	5 (7,4%)	0.55 (-1.64, -11.52 to 9.88)
Hospital readmission 30 days, n (%)		1 (2,9%)	3 (4,4%)	0.58 (-1.55, -8.92 to 5.81)
Overall mortality 30 days, n (%)		0	0	1
Surgery-related mortality 30 days, n (%)		0	0	1
Length of stay, median (IQR, range) days		3 (1, 3–54)	3 (1, 3–8)	0.27

L-TIA: Laparoscopic approach with totally intracorporeal anastomosis, R-TIA: Robotic approach with totally intracorporeal anastomosis, ICU: Intensive Care Unit, CRP: C-reactive protein, OR: Operating room, ED: Emergency department, IQR: Interquartile range

**Table 3:** Histopathological findings in TIA technique group patients

Characteristic	Laparoscopic-TIA (*n* = 35)	Robotic-TIA (*n* = 68)	*p*-value (95% CI)
Pathological T Stage, n (%)			0.21
pTis	1 (2,9%)	1 (1,5%))	
pT1	3 (8,8%)	11 (16,2%)	
pT2	6 (17,6%)	6 (8,8%)	
pT3	22 (64,7%)	37 (54,4%)	
PT4	2 (5,9%)	13 (19,1%)	
Pathological N Stage, n (%)			0.90
pN0	24 (68,6%)	45 (66,2%)	
pN1	6 (17,1%)	11 (16,2%)	
pN2	5 (14,3%)	12 (17,6%)	
Overall lymph nodes found, median (IQR)	19 (9)	18 (13)	0.68
*N* ≥ 12	34 (97,1%)	60 (88,2%)	0.13 (8.91, -0.53 to 18.35)
Maximum tumor diameter, median (IQR), cm	4 (2,5)	4,1 (2)	0.63
Postoperative UICC Stage, n (%)			0.89
I	9 (25,7%)	14 (20,6%)	
II	13 (37,1%)	27 (39,7%)	
III	11 (31,4%)	21 (30,9%)	
IV	2 (5,7%)	6 (8,8%)	

Laparoscopic-TIA: Laparoscopic approach with totally intracorporeal anastomosis, Robotic-TIA: Robotic Approach with totally intracorporeal anastomosis, UICC: Union for International Cancer Control, IQR: Interquartile range

### Comparative study between surgical techniques: EA technique group versus TIA technique group

No significant differences were found in the primary outcome anastomotic leakage (3,9% vs. 3,9%, *p* = 1 (95% CI: 0, -5.28 to 5.28), or anastomotic leakage with surgical treatment (1,9% vs. 1%, *p* = 0,5, 95% CI: 0.97, -2.30 to 4.24).

The rate of intraoperative events was higher in the EA group (*p* = 0.03). Twenty-four intraoperative complications or events were recorded in the EA group versus twelve in the TIA group (four in L-TIA and eight in R-TIA).

No differences were observed between the groups in terms of the type of intraoperative events that occurred. There were four failures of the air leak test, all in sigmoidectomies, and all were solved with reinforcement of the anastomosis with single stitches. Relevant bleeding in four (no transfusions were needed, and bleeding controlled during surgery), small bowel or colon detachment in five (no perforation), low-grade spleen laceration in four, and technical difficulties in three. The remaining cases were instances of dissection difficulty due to adhesions. Conversion to open surgery was higher in the EA group (9 cases vs. 0 cases, *p* = 0.002).

No significant differences were found in overall postoperative morbidity (37.9% vs. 29.1%, *p* = 0.14) or mortality (1 case vs. 0 cases, *p* = 0.5). This also applies to SSI (8,7% vs. 6.8%, *p* = 0.60) adynamic ileus rate (4.9% vs. 6.8%, *p* = 0.55), length of stay (median of 3 days in both groups), and the rate of reoperation (1.9% vs. 5.8%, *p* = 0.15).

Relevant anastomotic bleeding was higher in the EA group than in the TIA group (15.5% versus 6.8%, *p* = 0.04, 95% CI: 8.74, 0.22 to 17.26). A higher rate of return to ED was observed in the EA group (16.5% vs. 6.9%, *p* = 0.03, 95% CI: 9.71, 1.05 to 8.66). Significantly higher first- and third-day CRP levels were found in the TIA group (1st day CRP *p* ≤ 0.001, 3rd day CRP *p* = 0.008).

Only one patient died during the postoperative period, in the EA group, due to a sudden cardiorespiratory arrest 24 h after surgery. The autopsy revealed a large hemoperitoneum. It was not possible to establish whether this, or the resuscitation maneuver, was the cause of death.

As regards oncological outcomes, there were no significant differences in the tumor staging between groups (pT, *p* = 0.63, pN, *p* = 0.09 with a majority being staged as T3N0, and UICC stage II and III). The median tumor size was similar in both groups (3.5 cm vs. 4 cm, *p* = 0.16).

As for the lymphadenectomy, a mean of 22 nodes were removed in the EA group vs. 18 in each TIA group, with statistical significance (*p* = 0.01). There were no differences between the groups in terms of lymphadenectomies yielding more than 12 nodes (89.3% vs. 91.3%, *p* = 0.63) (Table 4, 5 and 6).

**Table 4:** Patients’ characteristics and perioperative data between anastomotic techniques

		Entire cohort			Propensity score matching analysis (ratio 2:1) Laparoscopic approach		
Characteristic		**EA technique group** (***n = 103)***	**TIA technique group** (***n = 103)***	**p-value** **(95% CI)**	**EA technique group** (***n = 68)***	Laparoscopic TIA technique group (***n = 34)***	**p-value** **(95% CI)**
Sex, male/female, n (%)		66 (64,1%)/37 (35,9%)	55 (53,4%)/ 48 (46,6%)	0.12 (10.68, -2.69 to 24.05)	39 (57,4%)/29 (42,6%)	18 (52,9%)/ 16 (47,1%)	0.67 (4.41, -16.07 to 24.9)
Age, median (IQR), years		69 (13)	71 (15)	0.68	69 (14)	68 (17)	0.67
BMI, median (IQR), kg/m2		27,39 (5,84)	27.4 (6,78)	0.63	27.12 (6.3)	26.46 (6.2)	0.99
ASA score (average), n (%)	I	4 (3,9%)	11 (10,7%)	0.12	3 (4.4%)	2 (5.9%)	0.94
	II	58 (56,3%)	47 (45,6%)		34 (50%)	15 (44.1%)	
	III	39 (37,9%)	40 (38,8%)		29 (42.6%)	16 (47.1%)	
	IV	2 (1,9%)	5 (4,9%)		2 (2.9%)	1 (2.9%)	
ASA score ≥ III, n (%)		41 (39,8%)	45 (43,7%)	0.57 (-3.88, -17.34 to 9.57)	33 (48.5%)	17 (50%)	0.88 (-1.47, -22.05 to 19.11)
Surgical technique	Left colectomy	17 (16,5%)	16 (15,5%)	0.22	14 (20.6%)	7 (20.6%)	0.98
	Sigmoidectomy	63 (61,2%)	53 (51,5%)		33 (48.5%)	16 (47.1%)	
	High anterior resection	23 (22,3%)	34 (33,0%)		21 (30.9%)	11 (32.4%)	
Previous abdominal surgery, n (%)		45 (43,7%)	49 (47,6%)	0.57 (-3.88, -17.48 to 9.71)	32 (47.1%)	17 (50%)	0.77 (-2.94, -23.51 to 17.63)
Operating time, median (IQR), min		165 (70)	205 (79)	≤ 0.001	160 (86.5)	185 (61.25)	0.052
Splenic flexure mobilization, n (%)		27 (26,2%)	30 (29,1%)	0.64 (-2.91, -15.12 to 9.30)	18 (26.5%)	9 (26.5%)	1 (0, -18.16 to 18.16)
Intraoperative complications, n (%)		24 (23,3%)	12 (11,7%)	0.03 (11.65, 1.40 to 21.90)	13 (19.1%)	4 (11.8%)	0.34 (7.35, -6.95 to 21.66)
Conversion to open surgery, n (%)		9 (8,7%)	0	0.002 (8.74, 3.28 to 14.19)	0	0	1

EA: Extracorporeal anastomosis, TIA: Totally Intracorporeal anastomosis, BMI: Body Mass Index, ASA: American Society of Anesthesiologists IQR: Interquartile range

### Propensity score matched analysis. EA technique group versus Laparoscopic-TIA technique group

After eliminating the robotic factor, we conducted a propensity score matching analysis within the laparoscopic approach to reduce confounding factors. Patients’ characteristics, operative method, perioperative, postoperative results, and oncological outcomes before and after the PSM are shown in Tables 4, 5 and 6.

**Table 5:** Thirty-day postoperative morbidity-mortality

		Entire cohort			Propensity score matching analysis (ratio 2:1) Laparoscopic approach		
Characteristic		**EA technique group** (***n = 103)***	**TIA technique group** (***n = 103)***	**p-value** **(95% CI)**	**EA technique group** (***n = 68)***	Laparoscopic TIA technique group (***n = 34)***	**p-value** **(95% CI)**
Overall morbidity, n (%)		39 (37,9%)	29 (28,2%)	0.14 (9.71, -53.07 to 22.48)	22 (32.4%)	12 (35.3%)	0.76 (-2.94, -22.48 to 16.6)
Clavien-Dindo classification (Cl-D)	0	64 (62,0%)	73 (70,9%)	0.43	46 (67.6%)	22 (64.7%)	0.34
	I	21 (20,4%)	14 (13,6%)		13 (19.1%)	5 (14.1%)	
	II	13 (12,6%)	10 (9,7%)		7 (10.3%)	3 (8.8%)	
	IIIa	0	0		0	0	
	IIIb	4 (3,9%)	6 (5,8%)		0	0	
	IVa	0	0		0	0	
	IVb	0	0		0	0	
	V	1 (1%)	0		0	0	
Low-grade morbidity (Cl-D ≤ II), n (%)		34 (33,0%)	24 (23,3%)	0.12 (9.71, -2.50 to 21.92)	20 (29.4%)	8 (23.5%)	0.53 (-5.88, -12.02 to 23.79)
High-grade morbidity (Cl–D > II), n (%)		5 (4,9%)	6 (5,8%)	0.75 (-0.97, -7.11 to 6.14)	2 (2.9%)	4 (11.8%)	0.09 (-8.82, -20.37 to 2.73)
Comprehensive Complication Index (CCI) score, median (IQR)		0 (8,7)	0 (8,7)	0.21	0 (8.7)	0 (8.7)	0.71
Surgical site infection (SSI), n (%)		9 (8,7%)	7 (6,8%)	0.60 (1.94, -5.36 to 9.25)	4 (5.9%)	4 (5.9%)	0.25 (-5.88, -18.07 to 6.31)
Incisional-SSI, n (%)		2 (1,9%)	1 (1%)	0.50 (0.97, -2.30 to 4.24)	0	0	1
Organ/space-SSI, n (%)		7 (6,8%)	5 (4,9%)	0.55 (1.94, -4.45 to 8.33)	4 (5.9%)	3 (8.8%)	0.42 (-2.94, -13.99 to 8.11)
Anastomotic leak, n (%)		4 (3,9%)	4 (3,9%)	1 (0, -5.28 to 5.28)	3 (4.4%)	2 (5.9%)	0.54 (-1.47, -10.76 to 7.82)
Surgical anastomotic leak, n (%)		2 (1,9%)	1 (1%)	0.50 (0.97, -2.30 to 4.24)	2 (2.9%)	1 (2.9%)	0.74 (0, -6.96 to 6.96)
Surgical complications, n (%)		32 (31,1%)	27 (26,2%)	0.44 (4.85, -7.48 to 17.18)	19 (27.9%)	10 (29.4%)	0.87 (-1.47, -20.13 to 17.19)
Non-surgical complications, n (%)		15 (14,6%)	8 (7,8%)	0.12 (6.80, -1.76 to 15.35)	9 (13.2%)	4 (11.8%)	0.55 (1.47, -12.03 to 14.97)
Post-operative ileus, n (%)		5 (4,9%)	7 (6,8%)	0.55 (-1.94, -8.33 to 4.45)	5 (7.4%)	3 (8.8%)	0.79 (-1.47, -12.85 to 9.9)
ICU admission, n (%)		1 (1%)	0 (0)	0.50 (0.97, -0.92 to 2.86)	0	1 (2.9%)	0.33 (-2.94, -8.62 to 2.74)
Relevant anastomotic bleeding, n (%)		16 (15,5%)	7 (6,8%)	0.04 (8.74, 0.22 to 17.26)	11 (16.2%)	2 (5.9%)	0.12 (10.29, -1.5 to 22.09)
Time to 1st meal, median (IQR), h		6 (0)	6 (0)	0.15	6 (0)	6 (0)	0.32
1st day CRP, median (IQR)		2,25 (2,04)	3.42 (3,79)	≤ 0.001	2.27 (1.99)	3.61 (5.01)	0.012
3rd day CRP, median (IQR)		2,76 (3,83)	4,11 (6,97)	0.008	3.1 (3.45)	4.1 (6.59)	0.17
Returns to OR, n (%)		2 (1,9%)	6 (5,8%)	0.15 (-3.88, -9.13 to 1.37)	1 (1.5%)	3 (8.8%)	0.10 (-7.35, -17.31 to 2.6)
Return to ED 30 days, n (%)		17 (16,5%)	7 (6,9%)	0.03 (9.71, 1.05 to 8.66)	8 (11.8%)	2 (5.9%)	0.28 (5.88, -5.13 to 16.89)
Hospital readmission 30 days, n (%)		5 (5,0%)	4 (3,9%)	0.74 (0.97, -4.61 to 6.55)	3 (4.4%)	1 (2.9%)	0.59 (1.47, -6.02 to 8.96)
Overall mortality 30 days, n (%)		1 (1%)	0	0.50	0	0	1
Surgery-related mortality 30 days, n (%)		1 (1%)	0	0.50	0	0	1
Length of stay, median (IQR, range) days		3 (1, 1–25)	3 (1, 3–54)	0.54	3 (1, 3–25)	3 (1, 3–54)	0.40

EA: Extracorporeal anastomosis, TIA: Totally Intracorporeal anastomosis, IQR: Interquartile range, ICU: Intensive care unit, CRP: C-Reactive protein, OR: Operating room, ED: Emergency department

**Table 6:** Histopathological findings

	Entire cohort			Propensity score matching analysis (ratio 2:1) Laparoscopic approach		
Characteristic	**EA technique group** (***n = 103)***	**TIA technique group** (***n = 103)***	**p-value** **(95% CI)**	**EA technique group** (***n = 68)***	Laparoscopic TIA technique group (***n = 34)***	**p-value** **(95% CI)**
Pathological T Stage, n (%)			0.63			0.23
pTis	0	2 (1,9%)		0	1 (2.9%)	
pT1	16 (15,5%)	15 (14,6%)		10 (14.7%)	4 (11.8%)	
pT2	9 (8,7%)	12 (11,7%)		6 (8.8%)	6 (17.6%)	
pT3	62 (60,2%)	59 (57,3%)		41 (60.3%)	21 (61.8%)	
PT4	16 (15,5%)	15 (14,6%)		11 (16.2%)	2 (5.9%)	
Pathological N Stage, n (%)			0.09			0.30
pN0	57 (55,3%)	69 (67,0%)		36 (52.9%)	23 (67.6%)	
pN1	30 (29,1%)	17 (16,5%)		21 (30.9%)	6 (17.6%)	
pN2	16 (15,5%)	17 (16,5%)		11 (16.2%)	5 (14.7%)	
Number of retrieved lymph nodes, median (IQR)	22 (13)	18 (11)	0.01	21.5 (13)	19 (9)	0.24
*N* ≥ 12	92 (89,3%)	94 (91,3%)	0.63 (-1.94, -10.02 to 6.14)	60 (88.2%)	33 (97.1%)	0.13 (-8.82, -18.36 to 0.71)
Maximum tumor diameter, median (IQR), cm	3,5 (2,5)	4 (2)	0.16	3.4 (2.5)	4 (2.6)	0.38
UICC Stage, n (%)			0.33			0.31
I	18 (17,5%)	23 (22,3%)		9 (13.2%)	7 (20.6%)	
II	32 (31,1%)	40 (38,8%)		24 (35.3%)	15 (44.1%)	
III	41 (39,8%)	32 (31,1%)		27 (39.7%)	11 (32.4%)	
IV	12 (11,7%)	8 (7,8%)		8 (11.8%)	1 (2.9%)	

EA: Extracorporeal anastomosis, TIA: Totally Intracorporeal anastomosis, UICC: Union for International Cancer Control, IQR: Interquartile range

No significant differences were found in the primary outcome anastomotic leakage (4.4% vs. 5.9%, *p* = 0,54), or anastomotic leakage with surgical treatment (2.9% in both groups *p* = 0.74).

Regarding perioperative variables, no differences are observed between groups in terms of operative time (160 min vs. 185 min, *p* = 0.052), intraoperative complications or events (19.1% vs. 11.8%, *p* = 0.34), or conversion to open surgery.

Concerning postoperative course, no differences were found in terms of postoperative morbidity (32.4% vs. 35.3%, *p* = 0.76) or mortality. Nor in other variables such as SSI (5.9% in both groups, *p* = 0.25), surgical complications (27.9% vs. 29.4%, *p* = 0.87) and non-surgical complications 13.2% vs. 11.8%, *p* = 0.55), post-operative ileus (7.4% vs. 8.8%, *p* = 0.79), relevant anastomotic bleeding (16.2% vs. 5.9%, *p* = 0.12), time to first meal (6 h in both groups, *p* = 0.32), return to OR (1.5% VS 8.8%, *p* = 0.10), return to ED (11.8% vs. 5.9%, *p* = 0.28), hospital readmission (4.4% vs. 2.9%, *p* = 0.59) and length of stay (3 days in both groups, *p* = 0.40).

The levels of PCR on the 1st postoperative day were significantly higher in the TIA group compared to the EA group (2.27 vs. 3.61, *p* = 0.012), but no differences were observed on the 3rd postoperative day (3.1 vs. 4.1, *p* = 0.17).

Regarding oncological outcomes, no differences were found between groups in terms of tumor staging ((pT, *p* = 0.23, pN, *p* = 0.30 with a majority being staged as T3N0, and UICC stage II and III), lymph node harvest (21.5 vs. 19, *p* = 0.24), percentage of patients with more than 12 nodes detected (88.2% vs. 97.1%, *p* = 0.13), or tumor size (3.4 cm vs. 4 cm, *p* = 0.38).

In the subgroup of obese patients, there were no differences in terms of postoperative complications (i.e., conversion to open surgery, anastomotic leakage, surgical site infections, relevant anastomotic bleeding, reoperation rate, hospital stay, or emergency department visits).

## Discussion

The minimally invasive approach for colorectal surgery, preferred for both oncological and benign cases, leads to fewer post-surgical complications, quicker recovery, less pain, and shorter hospital stays [1–4].

In right hemicolectomy, especially with a robotic approach, IA provides better short-term outcomes without adding risks [5, 6, 8, 44]. . However, anatomical complexities and anvil head insertion have limited IA use in left colon surgery.

Recently, TIA has been gradually incorporated into left colon surgery. Robotic approach has been a critical step in the evolution of TIA in recent years; it has helped to overcome the technical difficulties and has established a systematized and reproducible method for anastomosis.

Our comparative study did not show differences in patient demographic characteristics between groups.

In the preliminary analysis within the TIA group comparing the two approaches (laparoscopic and robotic), differences are only observed in terms of operative time, which is significantly longer in the robotic group. We interpret this result as both approaches being comparable in terms of outcomes, except for the operative time. The extended robotic surgery time is primarily due to technical factors like docking, instrument placement, and potential redocking as needed. The Propensity score matching analysis (PSM) demonstrate that laparoscopic EA and IA surgery is similar, implying that it doesn’t demand exceptional skills, and it suggest that the extended surgical time is primarily due to the robotic approach. For experienced colorectal surgeons, TIA isn’t a challenging procedure.

Regarding intraoperative variables, the operative time is not assessable, as we are aware that differences exist based on the approach. However, differences do appear in intraoperative complications and the conversion rate to open surgery. Based on this data, we can deduce that performing intracorporeal anastomosis reduces intraoperative negative events. However, the fact that patients with robotic approach are only in the TIA group and not in the EA group is an evident bias. When conducting the analysis exclusively with the laparoscopic approach, and thus eliminate the patients with robotic approach, the PSM shows us that there do not seem to be differences in any variables. This suggests that the robotic approach may be the key factor in reducing complications during the surgical process, but at the expense of a longer surgical time, which in communities with significant healthcare pressure is clinically relevant.

Concerning postoperative results, we observed a higher rate of anastomotic bleeding in the EA group compared to the TIA group. EA involves the extraction of the colon through the accessory incision, which increases mesenteric tension and may lead to ischemia, edema, delayed intestinal recovery or subsequent anastomotic bleeding. The rates of anastomotic dehiscence and adynamic ileus did not differ between the groups, but the rate of anastomotic bleeding was higher in the EA group. It results in a higher rate of visits to the emergency department, and it has an impact on health expenditure, and increases the overall healthcare costs per patient, which in turn influences clinical practice.

From a surgical perspective, there are no technical differences that could explain the variation in postoperative bleeding rates, aside from the need to extract tissue through an accessory incision to complete the resection and place the anvil head. In both the EA and TIA groups, using both approaches, the vascular division is performed at the same level (high ligation), and the colonic division is carried out mechanically with a linear stapler with the same caliber. Colorectal anastomosis is performed with the same circular stapler, also with the same caliber. No differences were observed in the number of linear stapler firings required for the colon resection.

Therefore, our hypothesis is that the tension exerted during the extraction of the sigmoid colon through the Pfannenstiel incision for resection and anvil placement can sufficiently damage the tissues, leading to a higher rate of postoperative bleeding at the anastomosis site compared to intracorporeal anastomosis, where such tension is absent. In addition, manipulation and exposure of the colon are reduced in the intracorporeal technique, minimizing tissue trauma and consequently, bleeding. Lastly, enhanced visualization and stability of the surgical field in intracorporeal laparoscopy may contribute to more effective hemostasis.

Eliminating the robotic factor in the PSM equalizes the outcomes, suggesting that the robotic approach plays a crucial role in minimizing unnecessary tissue manipulation, reducing anastomotic bleeding and consequently decreasing the rate of consultations to the emergency department.

If we review the literature on postoperative bleeding from the staple line, no differences are described between types of anastomosis (side-to-end or end-to-end) [18], nor in differences between robotic and laparoscopic approaches [19]. Regarding potential differences based on extracorporeal or intracorporeal anastomosis, most published comparative studies do not specifically describe this data [29, 30]. However, in studies that do describe it, no statistically significant differences are found [28, 32, 45]. It is noteworthy that these studies exclusively encompass patients treated with either laparoscopic or robotic approaches separately, thereby bolstering our hypothesis that the combined use of intracorporeal anastomosis and robotic approach constitutes a pivotal factor influencing the observed outcomes.

In relation to surgery-induced inflammation, limited literature explores the connection between CRP levels in laparoscopic and robotic approaches and their clinical relevance. In our study, the TIA group exhibited higher CRP levels on the first and third postoperative days. Within the laparoscopic approach group, the PSM analysis reveals higher levels of CRP on the first postoperative day, with no differences on the 3rd day.

There is limited literature describing the differences in surgical stress between different approaches and types of anastomosis in left colectomy. In the case of right colectomy, the results of the RCT conducted by Milone et al. comparing intracorporeal anastomosis (IA) with extracorporeal anastomosis (EA) show a reduced pattern of pro-inflammatory mediators (IL-6, CRP, TNF, and IL-1β) in patients with IA compared to those with EA, along with higher levels of anti-inflammatory cytokines. The results of the RCT conducted by Mari et al. showed similar findings, with lower levels of IL-6 and CRP in the IA group [46, 47].

For left colectomy, our results differ from those published by Widder et al. According to their findings, the levels of pro-inflammatory factors (leukocytes and CRP) are lower with the robotic approach [21].

These differences with the literature, with an increase in the inflammatory response in the TIA group, may be due to the longer operative time associated with the robotic approach. Nevertheless, this data does not correlate with an increase in postoperative complications or clinical impact, making it not clinically relevant.

In terms of oncological results, no differences are seen between both techniques. We only notice a higher number of nodes collected in the EA group (22) compared to the TIA group (18). However, both groups have a median greater than 12, with a percentage of patients having more than 12 nodes collected without differences, it does not seem to be clinically relevant. From an oncological perspective, it will remain pending to determine whether both techniques yield similar long-term oncological outcomes in terms of recurrence and survival.

Our findings align with other studies in literature. In the laparoscopic approach, recent retrospectives reveal IA’s advantages over EA, including reduced post-op complications, faster recovery, and shorter hospital stays. However, IA linked to longer operation times [27, 30]. In terms of surgical site infection, a multicenter propensity score-matched study found IA lowers the risk of both superficial and deep SSI [31].

IA has been associated with shortened hospital stay in some studies [30, 33], though others have not found significant differences. The same applies to operating time: IA is associated with a longer surgical time in most comparative studies, though the difference is not always significant [32]. 

Comparing laparoscopic and robotic approaches, recent studies show the robotic approach entails extended surgery time and higher costs [20–22]. However, systematic reviews and meta-analyses reveal lower rates of overall morbidity, anastomotic dehiscence, and SSIs (superficial and deep) in robotic surgery [19, 20].

The literature presents conflicting findings regarding the length of hospital stay. Some studies demonstrate a shorter hospital stay for robot-assisted surgery, while others indicate a longer stay, and the majority do not show any significant differences [21, 22].

In reference to oncological outcomes, our findings corroborate those of previous reports as far as the approaches were not associated with any significant differences. Only one multicenter study found robotic surgery to be associated with a higher number of harvested lymph nodes, but this increase did not affect recurrence, persistence, or 3- and 5-year survival rates [22]. 

IA takes longer with the robotic approach, but the rate of postoperative complications is lower [11, 19, 20]. IA appears to be associated with longer operative time, especially in patients with a BMI greater than 30. It also shows a lower conversion rate to open surgery and long-term hernias. However, no clear differences are evident regarding short-term postoperative complications (e.g., anastomotic dehiscence and surgical site infections), time to first meal, hospital stay, reoperation rate, or hospital readmission, although there was a slight trend towards improved surgical morbidity [26–28]. 

In general, the results obtained in our study are similar to those found elsewhere in the literature. They demonstrate a trend towards better postoperative outcomes with the creation of IA, especially using the robotic approach.

A debated concern is the possible rise in intra-abdominal infections from IA, linked to colotomy within the cavity and potential intestinal content spillage. In our study, all patients had antegrade colon preparation, mitigating contamination risk. Existing literature does not show elevated superficial or deep SSIs in IA for both right and left colectomies [6, 31].

Another matter of concern is the possibility of tumor dissemination due to colonic opening in oncological patients. To date, there are no reports of local or distant spread due to the creation of IA. However, since few studies on left colectomy have been performed, longer-term assessments are now needed to determine possible late complications.

This study has several limitations that should be discussed. The main limitation is the study design. It has been designed with a sample size calculation based on non-inferiority criteria, so the results may demonstrate its non-inferiority but not the superiority of the intracorporeal technique. Therefore, we can say that the results suggest better outcomes in some variables, but we cannot assert it statistically. This is a prospective (but not randomized) cohort study with a retrospective or historical control group, a factor that might cause some selection bias, which we have tried to mitigate by performing the propensity score matching analysis. Second, all surgeries were performed by a team of five surgeons, experts in both laparoscopic and robotic colorectal surgery. This limits its replication by non-expert teams. Third, the study has restrictive inclusion and exclusion criteria, which may affect the standardization of the technique: only cancer patients are included, excluding other pathologies such as diverticular disease or colon volvulus. The reason for this is to minimize the confounding effects of different pathologies on the results of the study. Patients with splenic flexure neoplasia were excluded since they may present different evolutions and complications compared to other locations in the left-sided colon, sigma, and upper rectum, and require different surgical procedures involving sectioning of other vascular branches, which might have affected the results.

Finally, our current goal is identifying subgroups that can maximize technique benefits based on anatomy or underlying factors (vascular issues, tumor location, nutrition, tumor size, surgical technique, etc.). Prospective controlled studies should now be carried out as the assessment phase (Stage III) in the IDEAL framework of surgical innovation.

## Conclusion

This study demonstrates that minimally invasive left colectomy with TIA, whether performed via laparoscopy or robot-assisted, is a feasible technique with satisfactory results compared to the standard technique with extracorporeal anastomosis. Based on the data obtained, we can conclude that the totally intracorporeal anastomosis technique is not inferior to the extracorporeal technique in terms of anastomotic dehiscence. The results indicate similar outcomes in terms of postoperative morbidity and other postoperative variables, with comparable pathological results. The robotic approach is poised to play a key role in improving short-term outcomes.

## Data Availability

No datasets were generated or analysed during the current study.

## References

[1] Tjandra JJ, Chan MK (2006) Systematic review on the short-term outcome of laparoscopic resection for colon and rectosigmoid cancer. Colorectal Dis. ;8(5):375 – 88. 10.1111/j.1463-1318.2006.00974. x. Erratum in: Colorectal Dis. 2008;10(3):305-6. PMID: 16684081 10.1111/j.1463-1318.2006.00974.x 16684081

[2] Lacy AM, García-Valdecasas JC, Delgado S, Castells A, Taurá P, Piqué JM, Visa J (2002) Laparoscopy-assisted colectomy versus open colectomy for treatment of non-metastatic colon cancer: a randomized trial. Lancet. ;359(9325):2224-9. 10.1016/S0140-6736(02)09290-5. PMID: 12103285 10.1016/S0140-6736(02)09290-5 12103285

[3] Bonjer HJ, Deijen CL, Abis GA, Cuesta MA, van der Pas MH; de, Lange-de Klerk ES, Lacy AM, Bemelman WA, Andersson J, Angenete E et al (2015) COLOR II Study Group. A randomized trial of laparoscopic versus open surgery for rectal cancer. N Engl J Med. ;372(14):1324-32. 10.1056/NEJMoa1414882. PMID: 25830422 10.1056/NEJMoa1414882 25830422

[4] Green BL, Marshall HC, Collinson F, Quirke P, Guillou P, Jayne DG, Brown JM (2013) Long-term follow-up of the Medical Research Council CLASICC trial of conventional versus laparoscopically assisted resection in colorectal cancer. Br J Surg 100(1):75–82. 10.1002/bjs.8945Epub 2012 Nov 6. PMID: 23132548 23132548 10.1002/bjs.8945

[5] Bollo J, Turrado V, Rabal A, Carrillo E, Gich I, Martinez MC, Hernandez P, Targarona E (2020) Randomized clinical trial of intracorporeal versus extracorporeal anastomosis in laparoscopic right colectomy (IEA trial). Br J Surg 107(4):364–372 Epub 2019 Dec 17. PMID: 31846067 31846067 10.1002/bjs.11389

[6] Allaix ME, Degiuli M, Bonino MA, Arezzo A, Mistrangelo M, Passera R, Morino M (2019) nov;270(5):762–767 Intracorporeal or Extracorporeal Ileocolic Anastomosis After Laparoscopic Right Colectomy: A Double-blinded Randomized Controlled Trial. Ann Surg. 10.1097/SLA.0000000000003519. PMID: 31592811 10.1097/SLA.0000000000003519 31592811

[7] Serra-Aracil X, Mora-Lopez L, Casalots A, Pericay C, Guerrero R, Navarro-Soto S, Hybrid NOTES (2016) TEO for transanal total mesorectal excision: intracorporeal resection and anastomosis. Surg Endosc 30(1):346–354 Epub 2015 Mar 27. PMID: 25814073 25814073 10.1007/s00464-015-4170-5

[8] Serra-Aracil X, Pascua-Sole M, Mora-Lopez L, Vallverdu H, Serracant A, Espina B, Ruiz C, Merichal M, Sanchez A, Romagnolo L et al (2020) Multicenter Controlled Study of Intracorporeal Mechanical Side-to-Side Isoperistaltic Anastomosis versus Extracorporeal Anastomosis in Laparoscopic Right Hemicolectomy: HEMI-D-TREND-Study. Dig Surg 37(4):271–274 Epub 2019 Oct 1. PMID: 31574504. on behalf of the HEMI-D-TREND-study group 10.1159/000502817 31574504

[9] Ceccarelli G, Biancafarina A, Patriti A, Spaziani A, Bartoli A, Bellochi R, Pisanelli MC, Casciola L (2010) Laparoscopic resection with intracorporeal anastomosis for colon carcinoma located in the splenic flexure. Surg Endosc 24(7):1784–1788. 10.1007/s00464-009-0853-0Epub 2010 Jan 1. PMID: 20044761 20044761 10.1007/s00464-009-0853-0

[10] Serra-Aracil X, Mora-Lopez L, Gomez-Torres I, Pallisera-Lloveras A, Serra-Pla S, Serracant A, Garcia-Nalda A, Pino-Perez O, Navarro-Soto S (2021) Minimal invasive surgery for left colectomy adapted to the COVID-19 pandemic: laparoscopic intracorporeal resection and anastomosis, a ‘don’t touch the bowel’ technique. Colorectal Dis 23(6):1562–1568. 10.1111/codi.15562Epub 2021 Feb 22. PMID: 33539644; PMCID: PMC8014247 33539644 10.1111/codi.15562 PMC8014247

[11] Serra-Aracil X, Mora-Lopez L, Gomez-Torres I, Pallisera-Lloveras A, Serracant A, Garcia-Nalda A, Pino-Perez O, Torrecilla A, Navarro-Soto S (2023) Laparoscopic and robotic intracorporeal resection and end-to-end anastomosis in left colectomy: a prospective cohort study - stage 2a IDEAL framework for evaluating surgical innovation. Langenbecks Arch Surg 408(1):135. 10.1007/s00423-023-02844-1PMID: 37002506; PMCID: PMC10065998 37002506 10.1007/s00423-023-02844-1 PMC10065998

[12] Mora LópezL, Pallisera Lloveras A, Serracant Barrera A, Garcia-Nalda A, Caraballo Angeli M, Pino Pérez O, Navarro Soto S, Serra Aracil X (2022) Robotic left hemicolectomy with intracorporeal anastomosis: description of the technique and initial results. Colorectal Dis 24(9):1080–1083. 10.1111/codi.16146Epub 2022 May 26. PMID: 35437870 35437870 10.1111/codi.16146

[13] Baker JW (1950) Low end to side recto sigmoidal anastomosis. Arch Surg 61:143–157 10.1001/archsurg.1950.01250020146016 15426554

[14] Akamatsu H, Omori T, Oyama T, Tori M, Ueshima S, Nishida T, Nakahara M, Abe T (2009) Totally laparoscopic low anterior resection for lower rectal cancer: combination of a new technique for intracorporeal anastomosis with prolapsing technique. Dig Surg 26(6):446–450. 10.1159/000239761Epub 2010 Jan 8. PMID: 20068315 20068315 10.1159/000239761

[15] Ohmura Y, Suzuki H, Kotani K, Teramoto A (2020) Intracorporeal hemi-hand-sewn technique for end-to-end anastomosis in laparoscopic left-side colectomy. Surg Endosc 34(9):4200–4205. 10.1007/s00464-020-07612-6Epub 2020 May 12. PMID: 32399939 32399939 10.1007/s00464-020-07612-6

[16] Benlice C, Aghayeva A, Yavuz E, Baca B, Hamzaoglu I, Karahasanoglu T (2019) Robotic left colectomy with complete mesocolic excision and intracorporeal side-to-side anastomosis for splenic flexure cancer with the Da Vinci Xi robotic platform - a video vignette. Colorectal Dis 21(12):1454. 10.1111/codi.14813Epub 2019 Aug 22. PMID: 31390679 31390679 10.1111/codi.14813

[17] Chierici A, Frontali A, Godefroy W, Spiezio G, Panis Y (2021) Can end-to-end anastomosis reduce the risks of anastomotic leak compared to side-to-end anastomosis? A comparative study of 518 consecutive patients undergoing laparoscopic total mesorectal excision for low- or mid-rectal cancer. Tech Coloproctol 25(9):1019–1026 Epub 2021 Jun 13. PMID: 34120290 34120290 10.1007/s10151-021-02468-x

[18] Habeeb TAAM, Mohammad H, Wasefy T, Mansour MI (2022) Outcomes of side-to-end versus end-to-end colorectal anastomosis in non-emergent sigmoid and rectal cancers: randomized controlled clinical trial. Ann Coloproctol. Mar 11. 10.3393/ac.2021.00906.0129. Epub ahead of print. PMID: 35272448 10.3393/ac.2021.00906.0129 PMC10338166 35272448

[19] Solaini L, Bocchino A, Avanzolini A, Annunziata D, Cavaliere D, Ercolani G (2022) Robotic versus laparoscopic left colectomy: a systematic review and meta-analysis. Int J Colorectal Dis 37(7):1497–1507. 10.1007/s00384-022-04194-8Epub 2022 Jun 1. PMID: 35650261; PMCID: PMC9262793 35650261 10.1007/s00384-022-04194-8 PMC9262793

[20] Lorenzon L, Bini F, Balducci G, Ferri M, Salvi PF, Marinozzi F (2016) Laparoscopic versus robotic-assisted colectomy and rectal resection: a systematic review and meta-analysis. Int J Colorectal Dis 31(2):161–173. 10.1007/s00384-015-2394-4Epub 2015 Sep 26. PMID: 26410261 26410261 10.1007/s00384-015-2394-4

[21] Widder A, Kelm M, Reibetanz J, Wiegering A, Matthes N, Germer CT, Seyfried F, Flemming S (2022) Robotic-assisted versus laparoscopic left hemicolectomy-postoperative inflammation status, short-term outcome and cost effectiveness. Int J Environ Res Public Health 19(17):10606. 10.3390/ijerph191710606PMID: 36078317; PMCID: PMC9517740 36078317 10.3390/ijerph191710606 PMC9517740

[22] Zheng H, Wang Q, Fu T, Wei Z, Ye J, Huang B, Li C, Liu B, Zhang A, Li F et al (2023) Robotic versus laparoscopic left colectomy with complete mesocolic excision for left-sided colon cancer: a multicenter study with propensity score matching analysis. Tech Coloproctol 27(7):569–578. 10.1007/s10151-023-02788-0Epub 2023 Apr 4. PMID: 37014449 37014449 10.1007/s10151-023-02788-0

[23] Cleary RK, Silviera M, Reidy TJ, McCormick J, Johnson CS, Sylla P, Cannon J, Lujan H, Kassir A, Landmann R et al (2022) Intracorporeal and extracorporeal anastomosis for robotic-assisted and laparoscopic right colectomy: short-term outcomes of a multi-center prospective trial. Surg Endosc 36(6):4349–4358. 10.1007/s00464-021-08780-9Epub 2021 Nov 1. PMID: 34724580; PMCID: PMC9085698 34724580 10.1007/s00464-021-08780-9 PMC9085698

[24] van Oostendorp S, Elfrink A, Borstlap W, Schoonmade L, Sietses C, Meijerink J, Tuynman J (2017) Intracorporeal versus extracorporeal anastomosis in right hemicolectomy: a systematic review and meta-analysis. Surg Endosc 31(1):64–77. 10.1007/s00464-016-4982-yEpub 2016 Jun 10. PMID: 27287905; PMCID: PMC5216072 27287905 10.1007/s00464-016-4982-y PMC5216072

[25] Tschann P, Weigl MP, Lechner D, Mittelberger C, Jäger T, Gruber R, Girotti PNC, Mittermair C, Clemens P, Attenberger C et al (2022) Is robotic assisted colorectal Cancer surgery equivalent compared to laparoscopic procedures during the introduction of a robotic program? A propensity-score matched analysis. Cancers (Basel) 14(13):3208. 10.3390/cancers14133208PMID: 35804985; PMCID: PMC9264883 35804985 10.3390/cancers14133208 PMC9264883

[26] Achilli P, Perry W, Grass F, Abd El Aziz MA, Kelley SR, Larson DW, Behm KT (2021) Completely intracorporeal anastomosis in robotic left colonic and rectal surgery: technique and 30-day outcomes. Updates Surg 73(6):2137–2143. 10.1007/s13304-021-01061-zEpub 2021 May 15. PMID: 33993462 33993462 10.1007/s13304-021-01061-z

[27] Hollandsworth HM, Li K, Zhao B, Abbadessa B, Lopez NE, Parry L, Ramamoorthy S, Eisenstein S (2022) Robotic left-stapled total intracorporeal bowel anastomosis versus stapled partial extracorporeal anastomosis: operative technical description and outcomes. Surg Endosc 36(5):3645–3652. 10.1007/s00464-022-09048-6Epub 2022 Jan 21. PMID: 35061081; PMCID: PMC9001240 35061081 10.1007/s00464-022-09048-6 PMC9001240

[28] Al Natour RH, Obias V, Albright J, Wu J, Ferraro J, Akram WM, McClure AM, Shanker BA (2019) Cleary R.K. A propensity score matched comparison of intracorporeal and extracorporeal techniques for robotic-assisted sigmoidectomy in an enhanced recovery pathway. J Robot Surg 13(5):649–656. 10.1007/s11701-018-00910-1Epub 2018 Dec 10. PMID: 30536133 30536133 10.1007/s11701-018-00910-1

[29] Brown RF, Cleary RK (2020 jun) Intracorporeal anastomosis versus extracorporeal anastomosis for minimally invasive colectomy. J Gastrointest Oncol 11(3):500–507. 10.21037/jgo.2019.12.02PMID: 32655928; PMCID: PMC7340812 10.21037/jgo.2019.12.02 PMC7340812 32655928

[30] Wang LM, Jong BK, Liao CK, Kou YT, Chern YJ, Hsu YJ, Hsieh PS, Tsai WS, You JF (2022) Comparison of short-term and medium-term outcomes between intracorporeal anastomosis and extracorporeal anastomosis for laparoscopic left hemicolectomy. World J Surg Oncol 20(1):270. 10.1186/s12957-022-02735-7PMID: 36030250; PMCID: PMC9419322 36030250 10.1186/s12957-022-02735-7 PMC9419322

[31] Guo Y, Li K, He L, Tong W, Chen Y, Wu B, Lin G, Qiu H, Xu L, Xiao Y, Wang Q (2023) Surgical site infection after intracorporeal and extracorporeal anastomosis in laparoscopic left colectomy for colon cancer: a multicenter propensity score-matched cohort study. Surg Endosc. May 11. 10.1007/s00464-023-10093-y. Epub ahead of print. PMID: 37170026 10.1007/s00464-023-10093-y 37170026

[32] Teramura K, Kitaguchi D, Matsuoka H, Hasegawa H, Ikeda K, Tsukada Y, Nishizawa Y, Ito M (2023) Short-term outcomes following intracorporeal versus extracorporeal anastomosis after laparoscopic right and left-sided colectomy: a propensity score-matched study. Int J Surg. May 24. 10.1097/JS9.0000000000000485. Epub ahead of print. PMID: 37222668 10.1097/JS9.0000000000000485 PMC10442079 37222668

[33] Swaid F, Sroka G, Madi H, Shteinberg D, Somri M, Matter I (2016) Totally laparoscopic versus laparoscopic-assisted left colectomy for cancer: a retrospective review. Surg Endosc. ;30(6):2481-8. 10.1007/s00464-015-4502-5. Epub 2015 Sep 3. PMID: 26335075 10.1007/s00464-015-4502-5 26335075

[34] Masubuchi S, Okuda J, Hamamoto H, Ishii M, Osumi W, Yamamoto M, Inoue Y, Tanaka K (2019) Uchiyama K. Intracorporeal Versus extracorporeal anastomosis in laparoscopic left colectomy for left sided Colon cancer: a retrospective study. Clin Surg 4:2506

[35] Pennell CP, Hirst AD, Campbell WB, Sood A, Agha RA, Barkun JS, McCulloch P (2016) Practical guide to the idea, Development and Exploration stages of the IDEAL Framework and recommendations. Br J Surg 103(5):607–615. 10.1002/bjs.10115Epub 2016 Feb 10. PMID: 26865013 26865013 10.1002/bjs.10115

[36] Hirst A, Philippou Y, Blazeby J, Campbell B, Campbell M, Feinberg J, Rovers M, Blencowe N, Pennell C, Quinn T et al (2019) No Surgical Innovation Without Evaluation: Evolution and Further Development of the IDEAL Framework and Recommendations. Ann Surg. ;269(2):211–220. 10.1097/SLA.0000000000002794. PMID: 29697448 10.1097/SLA.0000000000002794 29697448

[37] McCulloch P, Altman DG, Campbell WB, Flum DR, Glasziou P, Marshall JC, Nicholl J; Balliol Collaboration;, Aronson JK, Barkun JS et al (2009) No surgical innovation without evaluation: the IDEAL recommendations. Lancet. ;374(9695):1105-12. 10.1016/S0140-6736(09)61116-8. PMID: 19782876 10.1016/S0140-6736(09)61116-8 19782876

[38] Benson AB, Venook AP, Al-Hawary MM, Arain MA, Chen YJ, Ciombor KK, Cohen S, Cooper HS, Deming D, Farkas L et al (2021) Colon Cancer, Version 2.2021, NCCN Clinical Practice Guidelines in Oncology. J Natl Compr Canc Netw. ;19(3):329–359. 10.6004/jnccn.2021.0012. PMID: 33724754 10.6004/jnccn.2021.0012 33724754

[39] von Elm E, Altman DG, Egger M, Pocock SJ, Gøtzsche PC, Vandenbroucke JP (2007) STROBE Initiative. The Strengthening the Reporting of Observational Studies in Epidemiology (STROBE) statement: guidelines for reporting observational studies. Ann Intern Med. ;147(8):573-7. 10.7326/0003-4819-147-8-200710160-00010. Erratum in: Ann Intern Med. 2008;148(2):168. PMID: 17938396 10.7326/0003-4819-147-8-200710160-00010 17938396

[40] Crippa J, Calini G, Santambrogio G, Sassun R, Siracusa C, Maggioni D, Mari GAIMS, Academy Clinical Research Network (2023). ERAS Protocol Applied to Oncological Colorectal Mini-invasive Surgery Reduces the Surgical Stress Response and Improves Long-term Cancer-specific Survival. Surg Laparosc Endosc Percutan Tech. ;33(3):297–301. 10.1097/SLE.0000000000001181. PMID: 37184246 10.1097/SLE.0000000000001181 37184246

[41] Peel AL, Taylor EW (1991) Proposed definitions for the audit of postoperative infection: a discussion paper. Surgical Infection Study group. Ann R Coll Surg Engl ;73(6):385-8. PMID: 1759770; PMCID: PMC2499458 PMC2499458 1759770

[42] Horan TC, Gaynes RP, Martone WJ, Jarvis WR, Emori TG (1992) CDC definitions of nosocomial surgical site infections, 1992: a modification of CDC definitions of surgical wound infections. Infect Control Hosp Epidemiol 13(10):606–608 PMID: 1334988 1334988 10.1086/646436

[43] Amin MB, Greene FL, Edge SB, Compton CC, Gershenwald JE, Brookland RK, Meyer L, Gress DM, Byrd DR, Winchester DP (2017) The Eighth Edition AJCC Cancer staging Manual: continuing to build a bridge from a population-based to a more personalized approach to cancer staging. CA Cancer J Clin 67(2):93–99 Epub 2017 Jan 17. PMID: 28094848 28094848 10.3322/caac.21388

[44] Genova P, Pantuso G, Cipolla C, Latteri MA, Abdalla S, Paquet JC, Brunetti F, de’Angelis N, Di Saverio S (2021) Laparoscopic versus robotic right colectomy with extra-corporeal or intra-corporeal anastomosis: a systematic review and meta-analysis. Langenbecks Arch Surg 406(5):1317–1339 Epub 2020 Sep 9. PMID: 32902707 32902707 10.1007/s00423-020-01985-x

[45] Vaghiri S, Prassas D, Krieg S, Knoefel WT, Krieg A (2023) Intracorporeal versus extracorporeal Colo-colic anastomosis in minimally invasive left colectomy: a systematic review and Meta-analysis. J Gastrointest Surg 27(12):3024–3037. 10.1007/s11605-023-05827-1Epub 2023 Sep 12. PMID: 37698813; PMCID: PMC10837220 37698813 10.1007/s11605-023-05827-1 PMC10837220

[46] Milone M, Desiderio A, Velotti N, Manigrasso M, Vertaldi S, Bracale U, D’Ambra M, Servillo G, De Simone G, De Palma FDE, Perruolo G, Raciti GA, Miele C, Beguinot F, De Palma GD (2021) Surgical stress and metabolic response after totally laparoscopic right colectomy. Sci Rep 11(1):9652. 10.1038/s41598-021-89183-7PMID: 33958669; PMCID: PMC8102592 33958669 10.1038/s41598-021-89183-7 PMC8102592

[47] Mari GM, Crippa J, Costanzi ATM, Pellegrino R, Siracusa C, Berardi V, Maggioni D (2018) Intracorporeal Anastomosis Reduces Surgical Stress Response in Laparoscopic Right Hemicolectomy: A Prospective Randomized Trial. Surg Laparosc Endosc Percutan Tech. ;28(2):77–81. 10.1097/SLE.0000000000000506. PMID: 29360701 10.1097/SLE.0000000000000506 29360701

